# Marine Polysaccharides for Wound Dressings Application: An Overview

**DOI:** 10.3390/pharmaceutics13101666

**Published:** 2021-10-12

**Authors:** Shenghai Shen, Xiaowen Chen, Zhewen Shen, Hao Chen

**Affiliations:** 1 SDU-ANU Joint Science College, Shandong University, NO. 180 Wenhua West Road, Gao Strict, Weihai 264209, China; 201900700286@mail.sdu.edu.cn (S.S.); 201900700244@mail.sdu.edu.cn (X.C.); 2 The Key Laboratory of Synthetic and Biological Colloids, Ministry of Education, Jiangnan University, NO. 1800 Lihu Road, Wuxi 214122, China; 3 School of Humanities, Xiamen University Malaysia, Jalan Sunsuria, Bandar Sunsuria, Sepang 43900, Selangor, Malaysia; chs1909085@xmu.edu.my; 4 Marine College, Shandong University, NO. 180 Wenhua West Road, Gao Strict, Weihai 264209, China

**Keywords:** biopolymers, biomaterials, wound dressing, wound healing, chitosan, alginate, fucoidan, agar, carrageenan, ulvan

## Abstract

Wound dressings have become a crucial treatment for wound healing due to their convenience, low cost, and prolonged wound management. As cutting-edge biomaterials, marine polysaccharides are divided from most marine organisms. It possesses various bioactivities, which allowing them to be processed into various forms of wound dressings. Therefore, a comprehensive understanding of the application of marine polysaccharides in wound dressings is particularly important for the studies of wound therapy. In this review, we first introduce the wound healing process and describe the characteristics of modern commonly used dressings. Then, the properties of various marine polysaccharides and their application in wound dressing development are outlined. Finally, strategies for developing and enhancing marine polysaccharide wound dressings are described, and an outlook of these dressings is given. The diverse bioactivities of marine polysaccharides including antibacterial, anti-inflammatory, haemostatic properties, etc., providing excellent wound management and accelerate wound healing. Meanwhile, these biomaterials have higher biocompatibility and biodegradability compared to synthetic ones. On the other hand, marine polysaccharides can be combined with copolymers and active substances to prepare various forms of dressings. Among them, emerging types of dressings such as nanofibers, smart hydrogels and injectable hydrogels are at the research frontier of their development. Therefore, marine polysaccharides are essential materials in wound dressings fabrication and have a promising future.

## 1. Introduction

**Figure 1:**
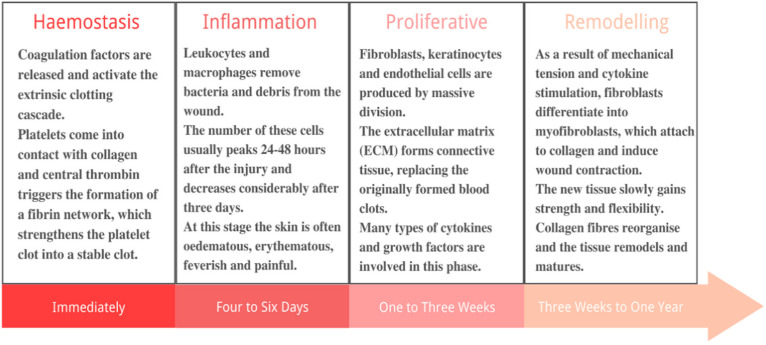
The four processes of wound healing.

Skin, being the largest organ of the human body, is the first immune barrier against external damage and invasion [1]. As a result, it is also one of the most frequently injured organs in the body [2]. There are two types of skin wounds: acute wounds and chronic wounds. Acute wounds usually heal within 1–12 weeks [3]. While chronic wounds are more susceptible to infection and require more healing time, bringing challenges for wound management. The degree of tissue damage and the organism’s tissue regeneration ability determine the repair mode and time. Wound healing is a complex process involving four steps: haemostasis, inflammation, proliferation and remodelling [4,5], as illustrated in Figure 1. Wound dressings have become a major wound healing treatment [6,7,8,9]. The ideal wound dressings should have the following characteristics: (1) to prevent further physical damage to the wound as a barrier to microbial invasion; (2) to ensure a certain degree of moisture on the contact surface between the dressing and the wound, providing a suitable environment for healing process; (3) to clear wound in time; (4) low adhesion to the wound to avoid secondary damage during dismantling; (5) good elasticity and gas permeability; (6) biocompatible, non-toxic and non-allergenic; (7) good haemostatic function, etc. [10,11,12,13,14]. Table 1 lists the advantages, disadvantages, and the suitable conditions of commonly used dressings.

Conventional dressings (e.g., gauze, bandages) could simply cover and protect the wound while failing to maintain a moist environment at the wound site. They have no direct effect on the wound with poor biocompatibility and may cause secondary injury when replaced or removed. Thus, they are believed unconducive to wound healing [13,24]. Comparatively, modern dressings interact with the wound and subsequently provide a more suitable environment for wound healing. Various polymeric wound dressings and coatings, such as polyurethane foam films and graphene dressings, have been well developed and widely utilized [25,26,27,28]. Nevertheless, the bioactivities and biocompatibility of these polymeric excipients are limited, which restricts their development. Therefore, natural polymers (e.g., polysaccharides, proteins) with good biocompatibility, biodegradability and similarity to the extracellular matrix (ECM) are widely advanced in wound dressings [29,30].

Polysaccharides are natural biopolymers that exist in various organisms. As a kind of essential macromolecular in life activities, polysaccharides are closely related to all types of biochemical metabolism [31]. Polysaccharide-based materials are widely used in wound dressings because they are non-toxic and biodegradable with colossal storage and good biocompatibility. The hydrophilic groups (carboxylic, amino, hydroxyl, and sulphate groups) in their structure can form non-covalent bonds with growth factors (GFs) to support bioadhesion. It is worth noting that many wound dressings with multiple activities can be prepared by simply processing the polysaccharide, for instance, by adding active substances, pairing copolymers, chemical modification, etc. Thus, natural polysaccharides have shown great application potential in wound management [32,33,34].

**Figure 2:**
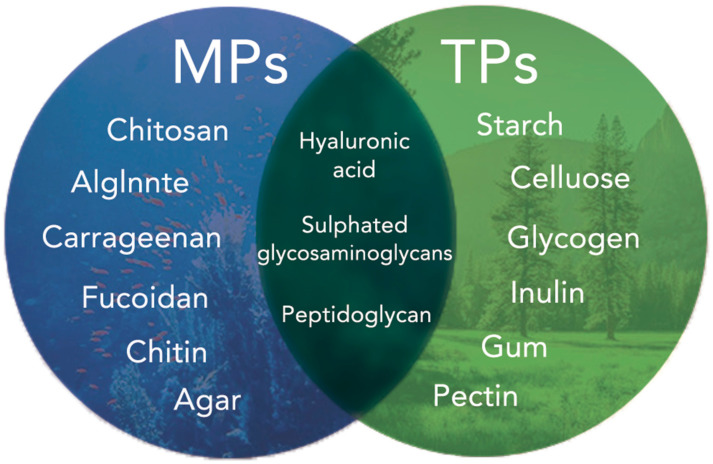
Classification of polysaccharides according to the source of extraction.

According to primary biological sources, polysaccharides can be classified into two main types: terrestrial polysaccharides (TPs) and marine polysaccharides (MPs) (Figure 2). MPs are one of the main components of all living marine organisms. Compared to TPs, MPs possess various properties that can be used for dressing development, such as antibacterial, antioxidant, anti-inflammatory, and so on [35]. In addition, most MPs have good histocompatibility, do not carry pathogens pathogenic to humans. With the advancement of biotechnology, the yield of MPs has increased dramatically, and the cost of extraction has decreased [36,37]. They are widely used to produce pure or complex polysaccharide-based biological preparations, such as hydrogels, membranes, and sponges. Moreover, they can also be used to make nanomaterials such as nanofibres and nanoparticles [35,36,37,38]. Therefore, MPs are promising biomaterials for the fabrication of wound dressings.

In this review, we focus on an overview of the application and enhancement strategies of marine polysaccharides in wound dressings. We first collected and analysed data from recent and ongoing studies to explain and illustrate the current research status of the development of marine polysaccharides. Subsequently, strategies for enhancing marine polysaccharide wound dressings are outlined, providing valuable information for wound dressings research. Lastly, we also discuss the research hotspots, intrinsic links, and development trends of MPs in wound dressings, and put forward the outlook for future research.

## 2. MPs for Wound Dressings

**Figure 3:**
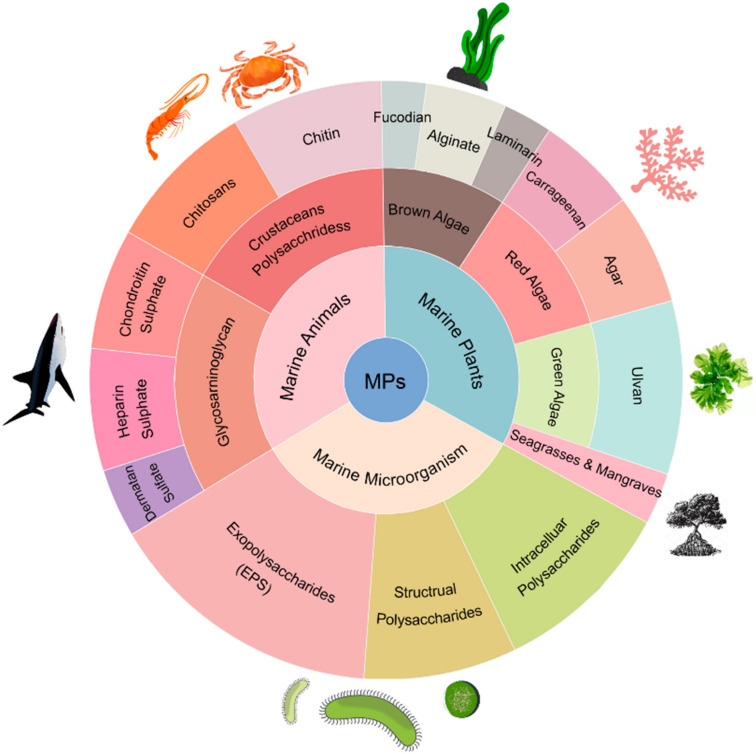
Classification of marine polysaccharides.

MPs meet the requirements for wound dressings materials, most of which are low-cost and easily accessible. Their highly biocompatible properties allow them to adhere to the skin without concern and be used in vivo. Moreover, they exhibit unique wound-healing activities. MPs can be divided into three main categories depending on the organism they originated from: marine animal polysaccharides (e.g., chitin, chitosan, marine glycosaminoglycans), marine algae polysaccharides (e.g., alginate, fucoidan, carrageenan) and marine microbial polysaccharides (Figure 3). The development of MPs wound dressings with different functions has become a hotspot. This paragraph elaborated the categorisation of MPs and the characteristics of the different MPs in wound dressings development.

### 2.1. Chitosan

**Figure 4:**
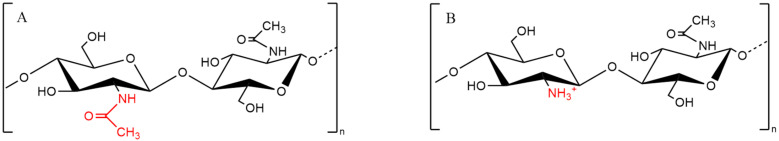
Chemical structure of chitin (**A**) and chitosan (**B**) fragments.

Chitin (Figure 4A), the second most abundant biopolymer in nature after cellulose, is a long and unbranched polysaccharide biopolymer composed of β-(1,4)-n-acetylamino glucose (GlcNAc). Chitin is mainly derived from the exoskeletons of marine crustaceans, such as shrimps and crabs [39,40]. Chitin is poorly water-soluble and not easily processed, whereas its derivative chitosan (CS) has a much wider application. CS is the only cationic polysaccharide among natural polysaccharides found so far (Figure 4B). CS can be formed by partial deacetylation of chitin under alkaline conditions [41]. When the degree of deacetylation reaches about 50%, CS dissolves in acidic aqueous solutions. After dissolution, the side chain amino groups of CS are transformed into cations that interact with other molecules. This is the reason why CS generates stable biomaterials with negatively charged polymers [42]. On the other hand, CS has a diverse range of modified derivatives. These derivatives have better solubility and bioactivities. Table 2 shows the most common CS derivatives.

CS and its derivatives are widely developed in wound dressings due to their ease of processing and multiple bioactivities. They are good gelling agents, and their cationic properties make them suitable for mixing with anionic copolymers to form hydrogels. Their good solubility and stability make them suitable for casting films, membranes and electrospinning into nanofibres when miscible with other compounds [47,48,49]. Furthermore, numerous amine groups of CS confer pH-dependent solubility, and its functional groups are suitable for Schiff base reactions and iminium reactions. This property provides CS with an advantage over other biomaterials for developing Smart hydrogels/injectable hydrogels/self-healing hydrogels [50,51,52]. Wound dressings using CS as starting materials have good physical properties for drug delivery. Many studies have shown that CS-based dressings can achieve sustained release and promote wound healing effectively [53,54,55]. Moreover, CS and its derivatives exhibit bioactivities that favour wound healing, such as antimicrobial, analgesic, antioxidant, anti-inflammatory, haemostasis, and promoting tissue regeneration. Table 3 shows the mechanism and characteristics of the bioactivities of CS for wound healing [56,57,58,59]. In addition, the hydrophilic group of CS allows their dressings to provide a moist healing environment for wounds [60]. These properties lead to CS dressings being a major part of MPs wound dressings.

Active agents carried by CS wound dressings could show a synergistic wound-healing effect with CS. This is due to the bioactivities of CS, which is dependent on cationicity (deacetylation degree) and its unique side chains structure, which has a mechanism that distinguishes it from other active substances [56,61]. The long-lasting bioactivities of CS can provide antibacterial properties at the end of the sustained release of loaded agents to prevent the recurrence of bacterial infections [62,63]. Furthermore, after the antibacterial/anti-inflammatory/antioxidant agents in the CS dressing improved the wound healing environment, CS regulates GFs to promote tissue regeneration and angiogenesis, effectively promoting wound healing [64,65]. Therefore, CS wound dressings can consistently optimise the four stages of wound healing synergistically with loaded agents, which is beyond the reach of most drugs and commercially available dressings [66].

Besides being a structural component for wound dressings, CS can also be added as an active agent. CS NPs are biocompatible and degradable. Their larger surface area allows for better use of the bioactivities of CS. They can be used as active agents directly embedded in hydrogels, membranes, and films, or as drug carriers for active agents to enhance activity [67]. The CS NPs embedded in the wound dressings allow for a double sustained release to reduce the cytotoxicity and resistance of the encapsulated drug while achieving sustained healing. In addition, due to the enhanced permeability of nanoparticles for superficial diffusion, CS NPs can treat infected wounds on a large scale and promote scar-free wound healing [68,69,70,71,72]. Another instance of using CS as an active agent is the CS coatings, which provided additional antibacterial, pro-healing activity to the dressing [73,74,75]. Therefore, the CS coating is also an approach for developing asymmetric membranes/multi-layer membranes dressings. Research has demonstrated that CS coatings provided antibacterial activity without affecting the structure of the original dressing and enhanced the overall mechanical properties of the dressing [76].

A large number of CS-based wound dressings have been commercialised, such as Celox™ [77], Chitopack C^®^, Chitoflex^®^ [78], Tegasorb^®^ [79], etc. The main forms of dressings include membranes, sponges, and hydrogels. These highly biocompatible dressings can be used for the management of acute/chronic wounds and, therefore, show great medical value [80].

Despite the fact that the development and productisation of CS wound dressings are now well advanced, a series of factors still hamper its development. A significant issue is the lack of prospective clinical trials. CS has been shown to be non-cytotoxic. However, its metabolic pathway in vivo is unknown, and there is a risk of cumulative toxicity [81]. Many recent studies have selected its derivative CMC to develop Injectable hydrogels as in vivo wound dressings [82,83]. The water-soluble CMC is free from cumulative and acute toxicity [84]. Most applications of CMC are still in the laboratory stage due to the difficulty of processing, but it has the potential to replace CS in the preparation of in vivo wound dressings in the future.

### 2.2. Marine Glycosaminoglycans

**Figure 5:**
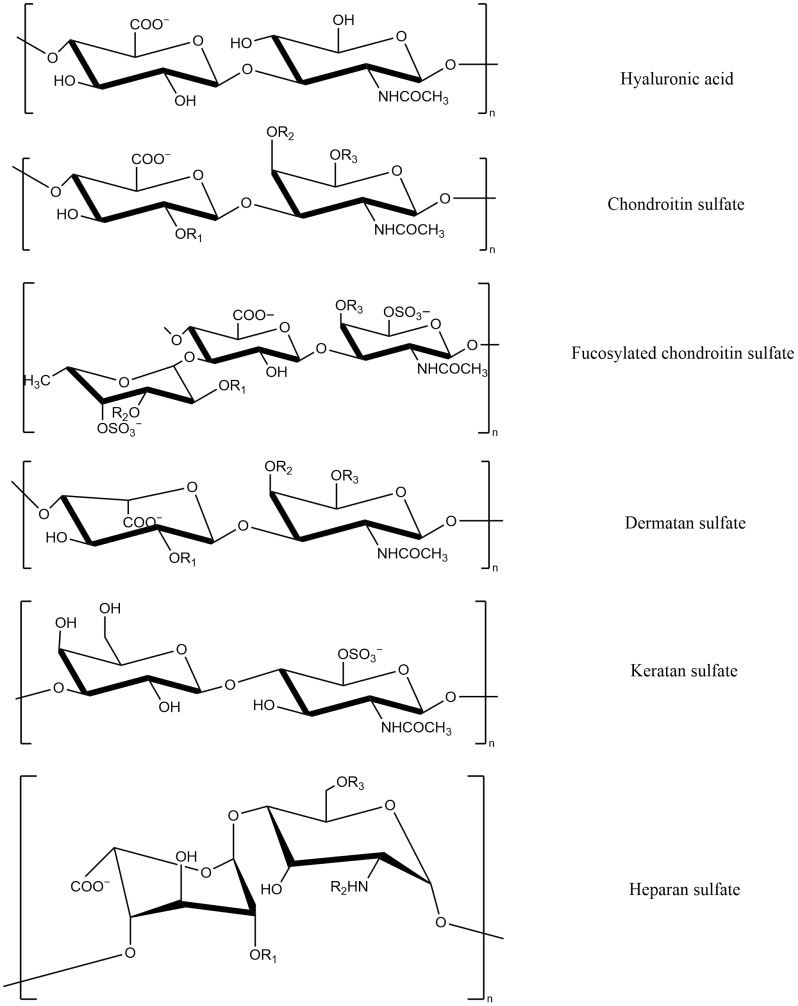
Chemical structures of common GAGs fragments.

Glycosaminoglycans (GAGs) are biopolymers consisting of repeating chains of O-linked disaccharide units commonly found in the ECM and on the cell surface of animal tissues (Figure 5) [85]. GAGs can be sulphated (chondroitin sulphate, skin-sulphate, heparin/heparin sulphate and dermatan sulfate) or not (hyaluronic acid) [86]. Hyaluronic acid (HA), widely found in the extracellular matrix, is a naturally occurring acidic GAG. HA plays an essential role in inflammation, angiogenesis, and tumour microenvironment formation, which is therefore widely used in tissue engineering, soft tissue fillers, wound dressings, and other biomedical applications [87,88,89,90,91]. Sulphated GAGs, such as chondroitin sulphate, heparan sulphate, are found in the tissues of terrestrial and marine animals (e.g., intestinal mucosa, lungs, blood vessel walls, skin, bones, etc.) [92,93]. GAGs of terrestrial origin have been extensively studied. In particular, heparan sulphate and chondroitin sulphate of terrestrial mammalian origin have important applications in wound dressings as pro-regenerative substances [94,95,96]. However, sulphated GAGs in marine animals have been shown to differ in composition, sulphation level and properties from those identified in terrestrial animals. Representative sources and characteristics of marine GAGs in recent years are shown in Table 4.

Marine GAGs have qualities that can be utilised in wound management. Compared to terrestrial GAGs, marine GAGs have no risk of spreading prions, making them more biosafe [97]. These MPs have bioactivities such as anti-inflammatory, antioxidant, tissue regenerating, etc. [98,99,100]. Furthermore, marine heparins have weakened anticoagulant activity, making them more suitable for wound dressings [85]. A few studies have also demonstrated the potential of marine GAGs to develop wound dressing scaffolds [101]. However, the different extraction sources did not result in significant differences in the bioactivities favouring wound healing. Due to the difficulty of extraction, GAGs used in wound dressings are mainly of terrestrial origin [102,103,104]. In the future, finding easy extraction methods and inexpensive sources is prominent in developing marine GAGs for wound dressings [105,106].

**Table 4:** Sources and characteristics of representative marine GAGs found in recent years.

GAGs Types	Sources	Properties and Applications	Refs
Heparan sulfate	*Amussium pleuronectus*	Anti-thrombin A more bio-safe source of heparan sulphate	[107]
Heparan sulphate	*Portunus pelagicus*	Highly attenuated anticoagulant activity Treatment of Alzheimer’s disease	[108]
Heparan sulfate	*Ascidian Phallusia nigra*	Low anticoagulant and antithrombotic activity Effective in preventing metastasis of cancerous tissue	[109]
Chondroitin sulfate	*Ludwigothurea grisea*	Anti-inflammatory Blocking cancer metastasis	[110]
Chondroitin sulfate	*Oncorhynch*	Promotes collagen fibre formation Anti-ageing	[99]
Chondroitin sulfate	*Raja clavata*	Cheap raw material cost	[111]
Chondroitin sulfate	*Echinodermata Ophiuroidea*	Promoting fibroblast growth factor 2-induced cell signalling	[112]
Dermatan sulfate	*Echinodermata Ophiuroidea*	Promoting fibroblast growth factor 2-induced cell signalling	[112]
Dermatan sulfate	*Mitsukurina owstoni* *Prionace glauca*	Neurite outgrowth-promoting	[100]

### 2.3. Alginate

**Figure 6:**
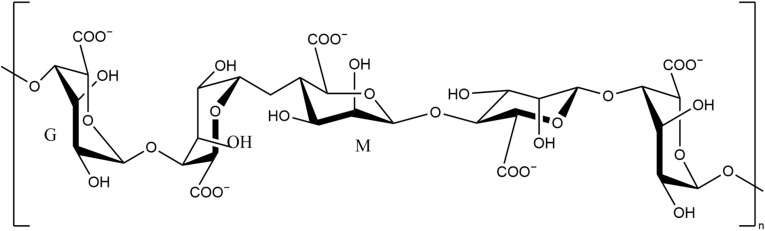
Chemical structure of alginate fragments.

Alginate (Figure 6) is a natural anionic biopolymer. It is a salt of alginic acid. Its molecule consists of different ratios of β-D-mannuronic acid (M) and α-L-glutamic acid (G), which determines its physical properties [2,113,114]. It is found mainly in the cell walls and intercellular mucus of brown algae and is also a source in some bacteria such as *Pseudomonas aeruginosa* and nitrogen-fixing bacteria. Due to its rheological properties, alginate has the advantage of thickening, stabilising, gel-forming, film-forming, fibre spinning, etc. [115,116].

Gel crosslinked by calcium, barium, and iron ions is the common form of alginate dressing [117,118]. Alginate can be used as a highly biocompatible inert carrier, thus exhibiting good drug delivery properties. Due to the presence of -COO-, alginate exhibits good adhesion in the targeted drug delivery pathways [119]. In addition, due to the high compatibility of alginate with human tissue, alginate dressings can be used as a barrier or as a drug carrier to treat mucosal tissue injuries that require long-term and better controlled drug delivery [119,120].

Wound dressings based on alginate are available in the form of hydrogels, films, and foams, etc. Numerous alginate dressings have been productised, such as Algicell™ [115], Guardix-SG^®^ [121], SeaSorb^®^ [121], Tromboguard^®^ [122], etc. Compared to conventional wound dressings, alginate dressings absorb wound fluids, form gels, maintain a physiologically moist environment, and minimize bacterial infections at the wound site.

Right now, some commercialized wound dressings are unable to maintain a moist environment, which is not only detrimental to wound healing, but also prone to cause difficulty in removing the dressing. The cross-linked G-chain of alginate could form a diamond-shaped pore containing a hydrophilic cavity, thus alginate would maintain and create a moist environment around the wound to promote wound healing [2,123,124]. In addition, due to its hydrophilic nature, alginate wound dressings could also rapidly absorb wound exudate and promote tissue repair. This property prevents the accumulation of exudate while preventing excessive dehydration of wounds. Therefore alginate dressings are favourable for severely exuding wounds [125,126].

Alginate also exhibits haemostatic and tissue regenerative activities. When in contact with wound exudate, it could accelerate blood coagulation due to the release of Ca^2+^ [127,128]. The high content of mannitic acid enabled alginate to induce cytokine production by human monocytes, thereby promoting tissue repair and enhancing chronic wound healing [121,129]. Furthermore, alginate dressings could promote angiogenesis, cell proliferation, and collagen deposition on traumatized surfaces [130,131,132]. This makes alginate dressings promising for developing dressings that promote tissue regeneration.

Nevertheless, there are still some limitations to the use of alginate wound dressings. When in contact with the physiological environment, alginate may gel instantly, preventing the bioactivities from taking effect [133]. Additionally, cations diffuse from regions of higher concentration to inner regions during cross-linking with cations, leading to a non-uniform distribution of alginate in the gel matrix network [134]. Thus, although alginate has shown its significant advantages as a wound dressing, it still has a wide range of development prospects.

### 2.4. Fucoidan

**Figure 7:**
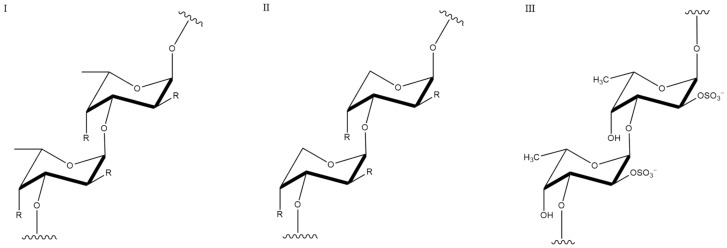
Chemical structure of fucoidan fragments (three structural types I, II and III).

Fucoidan (Figure 7) is a kind of sulphated polysaccharide widely distributes in the leaves of various types of brown algae (*Laminaria*, *Ascophyllum*, *Fucus*, etc.) and exoskeletons of some marine invertebrates. Fucoidan is composed of L-fucose, mannose, and glucose attached to a sulphate group. Its structure could be affected by harvest seasons and origins. *Fucus* and *Ascophyllumnodosum* contain mainly α (1→3) and α (1→4) fucoidan, whereas *Laminaria* contains mainly α (1→3) sulphate fucoidan [135].

Fucoidan has good antioxidant, antiviral, anticoagulant, anti-inflammation, antitumour and pro-regenerative activities. Recent studies have also shown that fucoidans have antibacterial activity, depending on their sulphation level [136,137,138,139]. Unlike CS and alginate, fucoidan is mainly adopted as the added active agent in wound dressing instead of primary substrates.

As a heparin analogue, fucoidan could modulate GFs. Early in 2004, O’Leary et al. demonstrated the ability of fucoidan to promote wound healing by increasing the rate of fibroblastic tissue regeneration [140]. Ozaltin et al. fabricated a modified polylactic acid scaffold loaded with fucoidan. The presence of fucoidan significantly increased cell proliferation and improved the cellular phenotype [141]. Sezer et al. combined fucoidan with CS to make a film to evaluate its therapeutic ability on burns. The results showed that fucoidan promoted dermal papillae re-surfacing and re-epithelialisation [142]. Wound dressings incorporating fucoidan exhibit a variety of abilities to optimise the healing process, including promoting collagen formation, promoting follicle regeneration, reducing inflammatory responses, reducing scar formation, and promoting angiogenesis [143,144,145,146].

Fucoidan and its derivatives are efficient in scavenging hydroxyl radicals and DPPH, exhibiting good antioxidant properties [147]. Park et al. found that low molecular weight fucoidan could reduce the lipid peroxidation of inflammatory cells [148]. Zeng et al. modified the CS with fucoidan and then combined it with alginate to form a GF-loaded scaffold dressing. The results demonstrated that 43% of sulphated fucoidan could scavenge DPPH and protect cells from ROS damage [149].

Since post-operative adhesions could often lead to chronic pain and various complications, developing anti-adhesive dressings on the surgical area is essential [150]. Many studies have implicated that dressings containing fucoidan effectively prevent tissue adhesions [151,152,153]. Fucoidan not only has anti-inflammatory properties but also antagonises the cytokine P-selectin, which mediates adhesion between endothelial cells and neutrophils [154]. Considering that many injectable MPs-based gels have been used to manage post-operative wounds, fucoidan anti-adhesive dressings have promising prospects for development.

Despite all these advantages, fucoidan dressings are not fully developed. This is mainly due to the unclear metabolic pathway of fucoidan and the risk of its accumulation in the liver and blood [155,156]. Clinical trials are already performed to test the toxicity of fucoidan to humans [135]. In the future, when its properties are fully understood, fucoidan may be used in a broader range of wound dressings.

### 2.5. Laminarin

**Figure 8:**
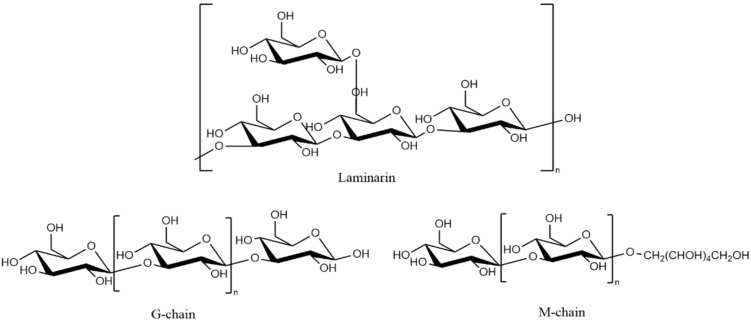
Chemical structure of laminarin fragments and two types of chains.

Laminarin (Figure 8) is a polysaccharide in the cell walls of brown algae (*Laminaria japonica, Ecklonia kurome*, etc.). Laminarin consists of β-glucan linked by (1,3) and (1,6) glycosidic bonds. Depending on the reducing end of the polysaccharide polymerisation chain, it can be divided into M-type chains with a 1-O-substituted D-Mannitol group and G-type chains ending in a D-glucose unit. The ratio of the two types of chains is influenced by the type of brown algae, the habitat, and the harvesting season, allowing laminarin to show different structures and bioactivities.

Laminarin has attracted much attention in recent years. The most notable activities of laminarin include anti-tumour, anti-inflammatory, immunostimulatory, antioxidant and anticoagulant activities [157]. Moreover, laminarin could induce angiogenesis and modulates GFs levels to promote tissue regeneration [158,159]. The relatively low molecular weight of laminarin makes it soluble in water and organic solvents, allowing them easy to process. [160]. Sellimi et al. found that creams with laminarin stimulated tissue regeneration and increased blood vessel density, thus effectively promoting wound healing [161]. Another study demonstrated that the addition of laminarin promoted cell adhesion and proliferation on the gel’s surface, enhancing the hydrogel’s wound treatment effect [162]. On the other hand, Kim et al. treated melanoma excision wounds with a dressing loaded with laminarin. Due to the antioxidant and anti-tumour activity of laminarin, the composite film promoted fibroblast growth, modulated apoptosis-inducing factors, and inhibited the proliferation of tumour cells [163]. This study showed the potential of laminarin dressings for the management of post-operative oncological wounds. These studies suggested the potential of laminarin in the development of hydrogel dressings.

As an emerging active substance, laminarin has not been used in the development of wound dressings yet. However, the various types of activity it has shown prove the great potential of this category of MPs.

### 2.6. Carrageenan

**Figure 9:**
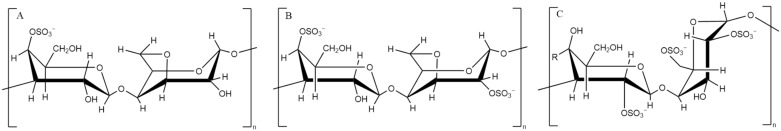
Chemical structure of carrageenan fragments ((**A**) Kappa; (**B**) Iota; (**C**) Lambda).

Carrageenan (Figure 9) is a hydrophilic colloid derived from red algae seaweeds *Kiringa, Stonecrop*, and *Deerstalker*. It is composed of alternating units of D-galactose and 3,6-anhydrogalactose (3,6-ag) linked with α-1,3 and β-1,4 glycosides. According to the forms of sulphate binding in them, they can be classified as K-type (Kappa), I-type (Iota) and L-type (Lambda) [164]. Carrageenan is extensively used in the pharmaceutical industry due to its gelling, thickening, and emulsifying properties.

Carrageenan gel is an excellent drug-loaded dressing with high elasticity and stability [165]. Thermal treatment and ionic crosslinking are the common means to induce carrageenan gelation. It can also cross-link with other polymers to design various hydrogel wound dressings [166,167]. The addition of carrageenan could significantly increase the stiffness, elasticity, and water retention of gels [168,169]. Additionally, the incorporation of nanoparticles or polymers into carrageenan gel could enhance its ability to absorb wound fluids and carrying drugs [170,171]. Carrageenan as an excipient could prolong the release of antimicrobial agents and growth factors [168,172]. The carrageenan injectable hydrogels could achieve continuous drug delivery to wounds [173,174]. Furthermore, other carrageenans micro-drug delivery systems (e.g., microspheres, pellets) have also been developed [171].

The similarity of the sulfated backbone structure of carrageenan to that of mammalian GAGs makes carrageenan-based wound dressings effective in promoting wound healing [170]. Carrageenan can change the porosity of the dressing, allowing nutrient transport and gas exchange across the wound healing site, activates the adhesion, diffusion, and proliferation of fibroblasts, enhances their differentiation capacity, promotes cellular transport to the injured skin, forms neovascularization, accelerates wound tissue repair and makes a significant contribution to wound healing [173,175,176]. The presence of many functional groups (such as hydroxyl and sulfate) in carrageenan, and its strong anionic properties make it easy to complex with other cations. The ion-carrageenan complex could promote the balance of anticoagulants and coagulation factors in the blood, making carrageenan an ideal material for promoting hemostasis [167,177]. Furthermore, oxidized carrageenan could inhibit the growth of Gram-positive and Gram-negative bacteria by disrupting bacterial cell walls and cytoplasmic membranes. [178,179].

On the other hand, too-high sulfate groups in carrageenan molecules might cause some detrimental effects on coagulation and the immune system [174]. Adjusting the sulfate groups through chemical modification, crosslinking, or incorporating biomolecules are the measures to enhance carrageenan safety. Therefore, carrageenan has a great potential for development in the preparation of wound dressings.

### 2.7. Agar

**Figure 10:**
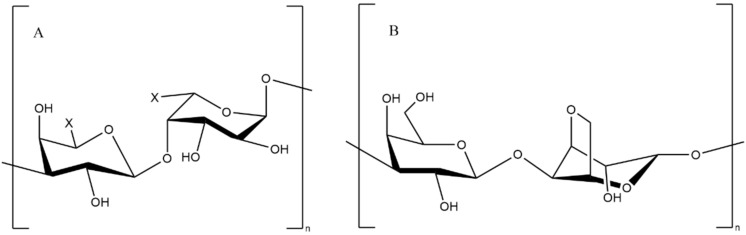
Chemical structure of agar (**A**) and agarose (**B**) fragments.

Agar is a kind of phycocolloid extracted mainly from red algae (such as *Lithospermum* and the *Gracilaria*) consisting of agarose and agaropectin (Figure 10). Agarose is an excellent gel-forming substance, which is responsible for the excellent physicochemical properties of agar gels. It consists of a disaccharide repeating unit consisting of 3-D-galactose and 4-linked 3,6-anhydro-1-galactose residues, with possible methoxy, sulphate, and other substituents in the polysaccharide chain. One of the features of agar is the significant temperature difference between its freezing and melting points. It needs to be heated to 95 °C before it starts to melt, and down to 40 °C before solidifying. This property makes adding active substances to agar gels easier than with other biomaterials [180,181]. Agar gels dressings are characterised by high-temperature resistance, high mechanical strength, and reversibility [182].

The gels prepared from agar are 2–10 times stronger than carrageenan, and the chemically modified ones have even higher mechanical strength [183]. The agar gel structure and properties are significantly dependent on its concentration. Guo et al. demonstrated that in composite membranes incorporating agar, the amount of agar is the main factor determining the physical properties of the membrane [184]. The highly absorbent feature of agar allows this composite hydrogel to absorb moisture to create a moist environment and promote wound healing [185]. On the other hand, agarose is almost entirely free of charged groups, which causes minimal denaturation and adsorption of sensitive biomolecules [186]. Additionally, the gel formation process of agarose is highly controllable [187]. The presence of agar/agarose would supply gels with high controllability of physical and chemical properties [188,189,190].

Agar-based gels are a promising drug delivery system because of their high and controlled drug loading capacity. The neutral surface charge and structural variability of agar gels make them efficient drug-loaded wound dressings [186]. Rivadeneira et al. adopted soy protein and agar to fabricate a composite membrane-embedded ciprofloxacin hydrochloride. The drug was released abruptly within the first 2 h, followed by a slow-release period of 2 weeks. Furthermore, the diffusive release period and amount of drug could be controlled by adjusting the agar content [191]. Agar gels also achieve high drug loading capacity while meeting proper mechanical strength and biocompatibility [192].

A small number of agar-based wound dressings are now commercially available, such as AgniGel^®^. Agar gels are highly biosafe and are used as inert carriers in most commercial dressings. Moreover, the biocompatibility and non-toxicity make agar an advantage over other materials used in the development of injectable hydrogels for in vivo wound management. Although the research is still in its infancy, the dressings that have been developed exhibit promising responsiveness and mechanical properties, demonstrating the great potential of this technology [193].

### 2.8. Ulvan

**Figure 11:**
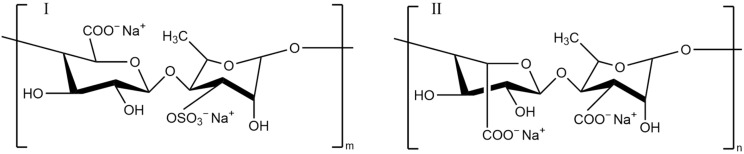
Chemical structure of ulvan fragments ((**I**) β-D-glucuronic acid (1->4) type; (**II**) α-D-glucuronic acid (1->4) type).

Ulvan (Figure 11) is a water-soluble sulphate heteropolysaccharide mainly found in the cell wall of *Ulva* genus green algae. It consists of rhamnose 3-sulphate, xylose-2-sulphate, glucuronic acid, and other polysaccharides. The ratio of these monosaccharide molecules in ulvan is highly variable and affects its physical and chemical properties [194]. The structure of the ulvan is influenced by the origin and season of collections. In addition, factors such as habitat and extraction conditions can also affect the fabric of the resulting ulvan. The bioactivities of ulvan depend mainly on its molecular weight, monosaccharide composition, and the content of sulfate and glyoxylate [195].

Rhamnose in ulvan modulates wound biosynthetic pathways and promotes tissue regeneration [195]. Since ulvan-gel is thermo-reversible, it could undertake controlled drug delivery. However, the water solubility of ulvan and the low mechanical strength of its gel limit the development of wound dressings [196].

Hydrophobic modification of ulvan is mostly utilized. Alves et al. modified ulvan by crosslinking it with 1,4-butanediol diglycidyl ether. The membrane has a significant water absorption capacity with suitable mechanical properties. In addition, the ulvan composite membrane achieves a burst-release of dexamethasone over 8 h and a sustained-release over a long period of 14 days [197]. This demonstrates the great potential of ulvan-based drug-loaded dressings. In the study by Chen et al., the hydrophobicity of ulvan was achieved by aromatization modifications. The modified ulvan was photocrosslinked to form a hydrogel. Ulvan’s activities allowed the hydrogel to improve cell survival and promote tissue regeneration [198].

In order to improve the poor mechanical properties, the preparation of ionic gels via the interaction of ulvan with cationic compounds is an effective method. CS-ulvan hydrogels were prepared by Mariia et al. using a lyophilization method. The cations of the CS side chains were able to react with the anions of the ulvan moiety to enhance stability. This composite hydrogel has good mechanical properties and provides a long-period sustained-release to promote wound healing [199]. Another way to improve the mechanical properties of ulvan dressings is to prepare ulvan nanofibres. The study by Kikionis et al. demonstrated the possibility of developing nanofibres by pairing ulvan with other polymers. These nanofibres are tough and have a long life span [200].

Green algae polysaccharides are not sufficiently developed for use in wound dressings. The difficulty of processing ulvan and its highly individual variability limit its application. Furthermore, clinical trials of ulvan are lacking [194]. However, modified ulvan still has great potential for wound dressings development. The search for an optimised carrier technology or an efficient way of chemical modification may be the method to develop ulvan further.

### 2.9. Marine Microorganisms Exopolysaccharides

Microbial polysaccharides are mainly water-soluble biopolymers, which can be divided into intracellular polysaccharides, structural polysaccharides, and exopolysaccharides (EPS). Compared with the first two, EPS have broader applications, as well as more comprehensive approaches to extract and process [201,202,203]. Many Gram-positive and Gram-negative bacteria, fungi and some algae could produce EPS [204]. The harsh environment of the ocean (an average depth of 3.8 km, pressure of 38 MPa, temperature of 2 °C, and other many extreme habitats) could induce marine microorganisms to produce unique EPS [205]. They could support microorganisms to tolerate biotic (e.g., competition) and abiotic stress factors (e.g., temperature, light intensity, pH, and salinity) [206]. Most EPS from marine microorganisms are heteropolysaccharides composed of various monosaccharides (including glucose, galactose, glucuronic acid, pyruvate, etc.) in a specific ratio [205,207,208,209,210].

Marine bacterial EPS have received a great deal of attention in recent years. EPS extracted from different microorganisms varied a lot [211]. Marine EPS have far more complex and diverse bioactivities than terrestrial EPS [201,205]. According to the previous research, EPS exhibit many properties that can be used in wound management, including antibacterial [212,213,214], antioxidant [215,216,217], anti-inflammatory [218,219], gel-forming [220], etc. In addition, several studies have reported that some marine microbial EPS could regulate wound cell metabolism to promote tissue regeneration and wound healing [215,221,222]. Table 5 presents several representative EPS.

Even though marine microorganisms EPS could provide various bioactivities, their utilization in wound dressings is still limited [230]. It could be mainly attributed to three main reasons. (1) The culturing, screening, and exploring specific marine microorganisms for EPS is a long-period study [231]. (2) The species diversity of marine EPS makes it challenging to process and costly to develop. This makes marine EPS unsuitable for developing wound dressings characterised by convenience and affordability [205]. (3) The bioactivities of marine EPS are not outstanding. Most of them provide only a limited type of wound healing activity and are no more active than other commonly used natural active substances. This means that they have no significant advantage as additional agents [232,233,234]. However, EPS such as xanthan gum can be produced commercially in large quantities [235,236]. Marine EPS has the potential to be used in large quantities in wound dressings if systematic production technologies can be developed for specific EPS-producing marine microorganisms.

## 3. Enhancement Strategies for MPs Wound Dressings

In order to enhance the therapeutic effect of MPs wound dressings and broaden their field of application, many enhancement strategies of wound dressings have been developed. These development strategies can be divided into two categories: (1) Enhancing the bioactivities (haemostatic, antibacterial, anti-inflammatory, etc.) of dressings; (2) Using the properties of different dressings or emerging dressing techniques to expand the range of applications.

### 3.1. Development of Activities-Enhanced MPs Wound Dressings

Adding active agents/polymers or modifying MPs to impart/synergise the bioactivities of MPs dressings is the main way to develop activities-enhanced dressings [56,237]. These activities are primarily used to accelerate and optimise the four stages of wound healing. Table 6 shows representative studies of activities-enhanced MPs wound dressings in recent years.

Haemostasis is the first stage of wound healing and a vital step in emergency medical care. Failure to haemostasis in time might lead to a lack of oxygen supply, subsequent damage to organs and even life-threatening conditions [277]. The haemostatic activity of MPs dressings is usually achieved by utilising the activity of MPs and their derivatives, as copolymers with other haemostatic materials, and by optimising the coagulation environment [60,278,279,280]. Some MPs, especially CS, alginate and carrageenan, have excellent haemostatic properties [173,281]. Through graft modification, the haemostatic properties of CS and its derivatives (e.g., quaternary ammonium CS and carboxymethyl CS) were enhanced [282,283]. CS could promote the local aggregation of clotting factors, red blood cells and platelets as well as accelerate the adhesion of blood components to their surface [284]. Combining with other haemostatic materials is another way of developing haemostatic dressings for MPs. The complex of gelatin-CS exhibited efficient haemostatic ability. This is due to the synergistic effect of their bioactivities, with gelatin increasing the number of platelets and leucocytes, while chitosan induces the release of clotting factors from platelets [285,286,287]. In addition, some MPs dressings are able to apply proper compression for the wound to enhance haemostasis property. This effect is mainly achieved by enhancing the adhesion of the MPs dressing. Adhesive dressings are applied tightly to the wound with compression to promote clotting, exemplified by the mussel-inspired technology [288,289,290]. In recent years, with the development of smart hydrogel technology, injectable thermosensitive MPs hydrogels have been widely explored for in vivo wound haemostasis applications. This emerging dressing has excellent potential for exploitation [189,291].

Another activity that needs to be provided from the haemostatic stage is antibacterial. Treating acute wounds without providing a means of antibacterial can easily lead to infection, preventing the formation of new blood vessels and tissue. This leads to an imbalance between the regulatory molecules involved in healing and thus hinders wound healing. It is also considered to be the most common factor affecting the deterioration of acute wounds into chronic wounds [292]. Some MPs, such as CS, have good antibacterial properties under acidic conditions. However, the antimicrobial properties of MPs are not sufficient as an antimicrobial dressing. In addition to preparing their derivatives (e.g., N, N, N-trimethyl CS chloride), another way is to add antimicrobial substances [293,294]. The most commonly added agents in current research of MPs wound dressings are metal nanoparticles (NPs), which are safer and more efficient than metal ions. Ag NPs are the most widely used metallic broad-spectrum antibacterial [295,296], with the rest including Au NPs [297], Cu NPs [298], ZnO NPs [299,300], AgSD NPs [301], CeO_2_ NPs [302] and TiO_2_ NPs [303]. These metal NPs show good inhibition against *E. coli*, *Pseudomonas aeruginosa*, *Staphylococcus aureus*, etc. [304]. Moreover, some studies have shown that metal NPs do not affect the mechanical properties of MPs dressings and can even enhance stability through ion chelation or interaction with the matrix as a filler [253,305]. Jiang’s study showed that dressings with controlled release ability could effectively reduce the cytotoxicity of metal NPs [306]. However, metal NPs show a weak antibacterial property at neutral pH, and heavy metals are not degradable, posing a risk to be delivered in vivo [305]. Natural antimicrobial agents have shown the advantage of high biosafety. Honey, essential oils, tannins, active amino acids and peptides, hesperidin, etc., have been widely used in recent years [65,292,307,308,309]. Some studies reported that the addition of Manuka honey to MPs dressings showed good antibacterial activity against *Staphylococcus aureus*, *Streptococcus pyogenes*, *Acinetobacter baumannii*, *Pseudomonas aeruginosa* and *Proteus mirabilis* [248,310]. In addition, honey could also form composite hydrogels or films with MPs exhibiting controlled physical properties [311]. For infection-prone wounds, it is necessary to use antibiotic-loaded dressings to provide strong antimicrobial properties. Antibiotics commonly used in MPs dressings include gentamicin, mupirocin, minocycline, vancomycin and lidocaine [53,312,313,314,315]. To avoid drug resistance, the provision of a controlled release hydrogel carrier is necessary. Thanks to the high processability and the structural properties of MPs hydrogels, MPs-based antibiotic hydrogels could achieve a stable and slow release [316,317].

Inflammation is the second stage of wound healing. Failure to reduce inflammation promptly might lead to the deterioration of chronic wounds [318]. The most common method to enhance the anti-inflammatory properties of MPs dressings is the addition of active substances. Representative substances include: curcumin, tannins, essential oils, leaf extracts etc. [257,257,319,320,321]. Curcumin is a polyphenolic substance extracted from plant turmeric. Several studies have demonstrated that curcumin could advance the expression of the anti-inflammatory factor such as IL-10, inhibit the expression of pro-inflammatory factors such as TNF-α and reduce the level of inflammation in wounds without affecting the properties and activities of MPs [322,323].

Timely removal of oxygen species reactive (ROS) from the wound surface is vital for the inflammatory stage. Moderate ROS could facilitate wound healing by stimulating cell migration and angiogenesis, but excess ROS would exacerbate the inflammatory response and impede wound healing, especially in chronic wounds [324,325]. Grafting of reducing chemical components for modification is a common method to improve the antioxidant activity of MPs wound dressings. Zhao et al. grafted polyaniline onto a quaternary CS backbone and synthesised quaternary CS-polyaniline (QCSP) with improved water solubility and antibacterial ability. A series of QCSP-based hydrogels were developed, and these injectable self-healing hydrogels exhibited up to 84% DPPH clearance, indicating that they have excellent antioxidant capacity [259]. Other graft modifications, including aniline tetramers, catechol, and various phenols, were also adopted to enhance the antioxidant activity of MPs [326,327,328]. Another way to confer antioxidant properties is to add active agents. By incorporating them into MPs dressings, highly biosafe antioxidant dressings could be produced. Colobatiu et al. incorporated plantain, arnica, marigold, forsythia, calendula and calendula extracts into CS films and achieved excellent antioxidant activity [21,329].

Proliferation is the crucial stage of wound healing and directly determines the quality of the new tissue regenerated and the integrity of the skin. In addition to providing wound management as described above, another noteworthy means of optimising the proliferative stage is to promote GFs such as transforming GF beta, platelet-derived GF, and interleukin-1 to accelerate wound repair and angiogenesis [56]. MPs wound dressing delivery systems could synergistically promote wound healing by modulating GFs [198,329,330]. Furthermore, controlled-release of GFs is necessary to prevent the inactivation of GFs on the wound surface [265]. The addition of natural active substances could synergistically accelerate wound healing by inducing the expression of genes to regulate angiogenesis, promote early wound granulation growth and collagen deposition. Another means of accelerating tissue regeneration is to create a moist, breathable external environment along with appropriate physical compression. MPs nanofibre mats have been shown to have good breathability and. MPs/co-polymer complex scaffold could provide a moist healing environment based on the hydrophilic moieties and structural domains [330,331]. Liu et al. used catechol-modified CS to create a continuous production of reactive oxygen bionic film. The continuous provision of the right amount of oxygen could induce cytokine release and collagen synthesis [332]. In addition, similar to haemostasis, shrinkable, highly adhesive MPs dressings can simultaneously promote healing through physical/physiological pathways. One such technology with great potential is responsive self-shrinking hydrogels that aid wound closure at an early stage [266]. A more effective treatment for tissue regeneration is the MPs dressing combined with stem cell exosome therapy. Exosomes are small vesicles of membrane secreted by cells containing complex RNA and proteins. Stem cell exosomes contain various functional proteins and cytokines that promote cell migration, cell differentiation, and angiogenesis [333,334,335]. Li et al. demonstrated that exosomes encapsulated in CS dressings promoted the migration of dermal fibroblasts and human dermal microvascular endothelial cells by regulating signal transduction pathways [336]. Wounds treated with exosome-carrying CS hydrogels prepared by Nooshabadi et al. showed 83.6% wound closure and a high degree of re-epithelialisation [337]. This suggests that MPs dressing carrying exosomes are good skin tissue engineering for treating severe wounds (full-thickness wounds, chronic wounds, etc.) [272,337,338].

Excessive deposition of collagen in the proliferative and remodelling stage would lead to scar formation. Scars are aesthetically displeasing, and in severe cases, might lead to physical deformities [339]. CS and its strongly cationic derivatives (e.g., CMC) have excellent scar inhibition and are the biopolymers commonly used to fabric scar-free wound dressings [58,273,340]. Moreover, *Aloe vera* (AV), a natural agent, is extensively applied to prevent scar formation by promoting cell growth and deep skin regeneration [341]. Due to its high biocompatibility and non-irritating properties, AV is often used in combination with MPs to develop wound dressings. Many studies have shown that MPs wound dressings incorporating AV enhance scar inhibition by promoting wound contraction and orderly deposition of collagen [58,275,342]. Other natural agents also have been shown to enhance collagen repair, reduce collagen deposition and accelerate healing by impeding the growth of gelatinous scar tissue, such as heparin, essential oils, silk etc. [343,344,345]. Scar-free healing mediated by the addition of GFs is also a common approach. GFs added to MPs can optimise the wound healing process by regulating fibroblast proliferation and migration, collagen synthesis, and skin remodelling to achieve scar-free [276,346].

### 3.2. Development of Different Forms of MPs Wound Dressings

Different dressing forms are suitable for different wounds [11]. Along with enhanced activities, selecting suitable dressing forms or applying advanced dressing technology are also practical enhancement strategies for MPs wound dressings.

#### 3.2.1. MPs Hydrogel

Hydrogels are three-dimensional, cross-linked network gels in which the liquid phase is water. They could provide moisture, promote wound healing, and remove necrotic tissue. Their high water content could reduce the temperature of wounds and relieve pain. As a soft and pliable biomaterial, hydrogels can be used in nearly all types of tissue wounds [81,347]. MPs hydrogels for wound dressings are biomaterials that exhibit high swelling properties and provide a moist helpful environment for wound healing [60,129]. On the other hand, the semi-open nature of gels gives them an excellent drug-carrying capacity. Additionally, emerging controllable or responsive hydrogels exhibit a more comprehensive range of applications. Table 7 shows representative studies of modified/emerging MPs hydrogels in recent years.

Hydrogel dressings prepared with single MP are prone to lack mechanical strength. The lack of strong support is detrimental to the final remodelling stage of wound healing and may lead to secondary injury and wound re-injury. Therefore, almost all MPs-based hydrogels have incorporated copolymers to improve mechanical strength. The copolymer could be divided into synthetic (such as PVA/PEG/PVP/PCL, etc.) and natural polymers (such as hyaluronic acid/gelatin/pectin/cellulose/starch/dextran/konjac glucomannan etc.) [53,268,301,330,354,362,363,364,365,366,367,368].

Besides enhanced mechanical properties, hydrogels dressings made from materials with self-healing properties have the most extended service lifespan. These self-healing properties mainly depend on the spontaneous reconstruction of internal bonds [369]. Chen et al. designed self-repairing CS-konjac glucomannan hydrogels based on Schiff base reaction. The hydrogels repaired rapidly and showed excellent durability [370]. Ding et al. prepared interpenetrating polymer network (IPN) hydrogels by combining acrylamide-modified chitosan with oxidized alginate and polyvinyl alcohol (PVA) complex. The hydrogels showed excellent mechanical properties and good self-healing ability [371].

Smart Hydrogels based on MPs are an emerging type of wound dressings. Smart hydrogels can change their structures or chemical properties depending on intrinsic factors (e.g., time) or external stimuli (e.g., temperature/pH/light). Smart hydrogels are cutting-edge technology used in recent years to achieve the controlled release of agents and targeted wound therapy [372]. The thermosensitive hydrogel could form rapidly to cover the wound surface at body temperature makes them suitable for in vivo wound therapy. As the pH values of wounds generally vary from normal tissue, pH-sensitive MPs hydrogels can provide precise wound treatment. These gels release less drug in normal tissues with neutral pH, while the gel network voids become larger at alkaline or acidic pH, accelerating the drug release [301]. These hydrogels could achieve targeted drug delivery [249,373]. Wang et al. prepared a dodecyl-modified CS hydrogel equipped with a photothermal agent and an antibacterial drug. The hydrogel generated a large amount of heat and released the drug on demand under the irradiation of near-infrared light, achieving good antibacterial and antioxidant effects [353].

Injectable hydrogels (Injectable hydrogels) is achieved by injection of gel precursor and the aqueous solution of bioactive agents, which forms gels in the body [374]. Injectable hydrogels are formed in situ, meaning they can be used for the precise delivery of drugs to treat irregular, hard-to-reach wounds. Due to their biocompatibility, degradability and unique delivery method, MPs Injectable hydrogels have received considerable research in recent years. They have been used to treat post-operative wounds, joint wounds, full-thickness defects, and others that cannot be treated with conventional dressings [303,375,376,377]. Furthermore, MPs injectable hydrogels are excellent carriers for the sustained release of various cytokines and GFs due to the ease of adding active substances in the sol form. Various MPs Injectable hydrogels carrying regulatory factors such as basic fibroblast GF, stromal cell-derived factor-1 and vascular endothelial GF have been developed. These gels could provide accurate wound coverage and achieve sustained release, thus promoting tissue regeneration and accelerating wound healing [355,378,379,380].

Mussel-inspired hydrogels have been developed to mimic the adhesion mechanism mediated by marine mussel adhesion proteins. These hydrogels have far more powerful wet adhesion and mechanical properties than conventional hydrogels and can be used in a liquid environment [381,382]. Dopamine is structurally similar to mussel proteins and is most commonly used in developing mussel-activated hydrogels because of its ability to produce the active polymer dopamine (PDA) during oxidation [381]. Thanks to the bioactivities of MPs, the mussel-inspired MPs hydrogel has rapid haemostatic properties and promotes wound healing synergistically with the compression effect of the gel on the wound. MPs’ biocompatibility allows these emerging hydrogels to be used for in vivo wound management [238,358,359,383].

#### 3.2.2. MPs Nanofibrous

Nanofibres are wire-like materials with a certain aspect ratio at the nanometer scale. In recent years, electrospinning has become a core technology for the manufacture of nanofibres. The presence of repulsive forces between the charged groups of MPs complicates their electrospinning properties, while the resulting nanofibres have poor mechanical properties and degrade rapidly [384]. Other synthetic/natural polymers should be added to improve the stability of MPs nanofibres. Currently, the leading MPs used to develop nanofibres are CS and alginate. A representative application of MPs nanofibres in wound dressings is nanofibre mats (scaffolds), which can be further processed into nanofibre hydrogels, nanofibre membranes and other nanocomposite dressings. Since nanofibres are similar to ECM, nanofibre dressings can promote cell adhesion and proliferation, thereby facilitating wound healing [385,386]. Furthermore, the porous nanostructure allows for a uniform and robust distribution of the drug on the MPs nanofibrous scaffold, resulting in high drug loading, high encapsulation rates and prolonged sustained release properties [387,388]. The porous structure also allows for good breathability, facilitating wound healing [389]. Table 8 presents a summary of MPs nanofibre dressings in recent years.

#### 3.2.3. MPs Film/Membrane

Compared to 3D-structured hydrogels, films are often considered as 2D dressings, covering wounds flat and more acceptable to the patient. MPs-based membranes can be divided into traditional and nanofibre membranes (electrospun membranes). Traditional membrane dressings are thick and usually made through the casting process [402,403]. While electrospun membranes are thin and prepared by shaping nanofibre mats. Table 9 presents a summary of MPS-based membranes dressings in recent years.

The electrospun membrane has good mechanical properties and tissue regeneration ability. MPs electrospun membranes are porous and highly hydrophilic, thus could promote the adhesion and proliferation ability of fibroblasts. This could accelerate tissue regeneration and wound healing significantly [270,413]. Simultaneously, MPs nanofibre membranes exhibit appropriate water vapour transport and exudate absorption capacity, providing a suitable healing environment for the wound [405,414].

The planarized form of MPs-based membrane provides the multilayer design possibility. Asymmetric and multilayer membrane-based techniques are the most commonly used for MPs-based membrane dressings. Both types of technology provide better results by mimicking the natural skin structure. The outer side of the MPs-based asymmetric membrane generally provides protection, and the inner side provides bioactivities. This unique configuration gives it a better healing effect and offers therapeutic potential for wounds in complicated environments [415,416]. Hydrophobic substances could provide a hydrophobic and asymmetric outer surface for MPs membranes. The resulting membrane exhibits water-repellent protective, antibacterial and healing-promoting properties. This feature ensured the efficiency of the membrane dressing in wet and adverse environments [406,408]. The asymmetric membrane covered with a dense layer, on the other hand, has extremely high mechanical properties and achieves better resilience. The inner active layer can provide constant and stable wound treatment in an unaffected environment [390,417]. MPs multilayer films are considered to be the dressing that enables versatile and efficient drug delivery. Furthermore, the spatially designed structure of the multilayer membrane optimises the function of the components and provides a more suitable microenvironment, giving them a better wound healing capacity [412,418].

#### 3.2.4. MPs Sponge

MPs sponge is biodegradable and has good swelling properties to absorb wound exudate effectively. The porous and fluffy structure of the MPs sponge is ideal for acute and haemorrhagic wounds [419,420]. Wang et al. found that sponges had better water absorption, breathability, haemostatic properties and more remarkable pro-healing ability than hydrogels and membranes of the same composition (CMC) [421]. It is worth mentioning that CS-based sponge dressings are the efficient and widely adaptable biomaterial for haemostasis. This is since the sponge dressing has good blood-absorbing properties and fills the wound when swollen. The compression provided by the sponge works synergistically with the bioactivity of CS to haemostasis and effectively manage acute wounds [243]. Table 10 presented a summary of MPs-based sponge dressings in recent years.

#### 3.2.5. Other Types of MPs Dressings

Microspheres are organic or inorganic spherical free-flowing particles with diameters from 1 to 1000 μm that could encapsulate drugs [431]. Microspheres enable targeted drug delivery, controlled release and prolonged drug delivery. MPs microspheres dry powder can be used for wound management as it can indirectly act as a wound dressing by forming a hydrogel with absorbed wound exudate [67,432]. Romic et al. demonstrated that MPs microspheres have a sustained-release effect and inhibit common bacteria [433]. On the other hand, MPs microspheres can also be embedded in dressings as drug carriers providing sustained-release properties. Hydrogels carrying AgSD-loaded CS microspheres can effectively treat infected full-thickness wounds [434]. Alginate microspheres showed good loading efficiency and could improve wound healing [435].

Aerogel is a porous, ultra-lightweight material with high mechanical strength [436,437]. The large specific surface area of aerogels allows for better utilisation of the bioactivities of MPs molecules [438]. Recent studies have shown that CS-based aerogels have excellent haemostatic, antibacterial and growth-promoting activities [439,440,441]. Alginate based aerogels have high exudate absorption and the ability to bind therapeutic substances to promote wound healing, allowing for effective drug delivery to the wound [442,443]. The aerogel dressings are also patient-friendly, with less pressure and discomfort on the wound.

## 4. Conclusions

Marine polysaccharides are novel biological sources for wound dressings. The excellent biocompatibility and biodegradability make them suitable for tightly fitting to the skin. The diverse bioactivities provide excellent wound management and accelerate wound healing. Meanwhile, their low price is in line with the requirements of wound dressings.

Chitosan and alginate are two of the most important marine polysaccharides widely used in wound dressings. They can be used as starting materials, combined with other polymers or active agents, then processed into wound dressings such as hydrogels, membranes, films, nanofibres and sponge. In order to improve the effectiveness of wound therapy, various enhancement strategies have been used to develop these dressings with enhanced antibacterial, antioxidant, anti-inflammatory, pro-regenerative, scar-free activities. On the other hand, advanced and emerging dressing technologies have expanded the range of applications for these wound dressings, with technologies such as smart hydrogels, asymmetric/multi-layer films and nanofibre mats widely appearing at the forefront of marine polysaccharide wound dressing discovery. Although the number of studies on fucoidan, carrageenan, agar and ulvan is small, the dressings developed from them have interesting activities and can treat specific wounds. Some marine polysaccharides, such as laminarin, marine glycosaminoglycans and marine microbial EPS, are currently not or rarely used for wound management. However, these polysaccharides have also shown the ability to promote wound healing, suggesting that they have the potential to be developed into wound dressings.

Over the past few decades, the value of these natural macromolecules, once regarded as waste, has been gradually recognised. Research in the biomedical field based on marine polysaccharides has also become a hotspot. As clinical trials are improved, dressing technology is enhanced, and the extraction process is optimised, various marine polysaccharide wound dressings can be used in a broader range of medical applications.

## Figures and Tables

**Table 1:** Advantages, disadvantages, and the suitable conditions of dressing forms in modern medicine.

Dressing Types	Advantages	Disadvantages	Suitable Conditions	Refs
Hydrogels	Good absorption of exudate Good moisturizing properties Have a cleansing effect No reoccurring mechanical damage Self-adhesive Concealed appearance Good antibacterial properties Accelerated wound healing	Poor ability to absorb exudate Higher costs Possible allergic reaction	Pressure ulcers Surgical wounds Burns Radiation dermatitis Diabetic foot ulcer	[15,16]
Nanofibre mats	Good antibacterial properties Effective control of local wound infection Good absorption of exudate Accelerated wound healing	Cytotoxic risk Prone to allergic reactions Higher production cost	Burns and scald Localized trauma infection	[17,18]
Films	Good antibacterial properties Good moisturizing properties Self-adhesive	Poor mechanical properties Higher costs	Epithelializing wounds and superficial wounds with limited exudate Chronic venous ulcer Radiation dermatitis	[19]
Membranes	Good haemostatic effect Promotes granulation tissue formation and self-decomposition of necrotic tissue Good antibacterial property	Poor ability to absorb ooze Higher production cost	Chronic venous ulcer All kinds of dermatitis and eczema	[20,21]
Sponge	Good absorption of exudate Low permeability Good antibacterial properties Thermal insulation	Excessive absorption Higher costs Inconvenient to observe	Infected wounds Diabetic foot ulcer Medium to heavily exuding wounds Venous ulcers	[22,23]

**Table 2:** The most common CS derivatives used in biotechnology development.

Derivatives	Structures	Properties	Refs
Carboxymethyl chitosan	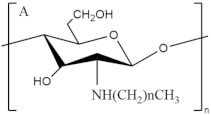	Better and more controlled water solubility Inhibits scarring	[43]
Alkylation chitosan	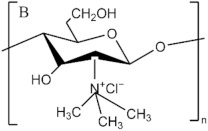	Better water solubility Enhanced haemostatic efficacy Better mechanical stability	[44,45]
Trimethyl chitosan ammonium	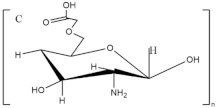	Water-soluble over a wide pH range Good flocculation and antistatic properties Better antibacterial properties	[46]

**Table 3:** Bioactivities of CS that facilitate wound healing and their mechanisms.

Bioactivities	Mechanisms and Hypotheses
Antibacterial	No definitive conclusion yet. The main hypotheses include: (1) adheres to and electrostatically disrupts bacterial cell walls and cell membranes, (2) chelates trace metal cations leading to potential imbalance, (3) interaction with intracellular targets to inhibit protein synthesis, (4) deposits on bacteria and affects metabolism
Anti-inflammatory	Induces increased levels of anti-inflammatory cytokines such as IL-10, TGF-β1 and decreased levels of pro-inflammatory cytokines.
Antioxidant	It is achieved by donating hydrogen atoms. The amino and carboxyl groups of CS stabilize free radicals.
Promotes tissue regeneration	Modulates growth factors to: promote macrophage transfer to wounds; promote fibroblast proliferation; promote proteoglycan and collagen synthesis; promote angiogenesis.
Haemostasis	Promotes the aggregation of platelets and red blood cells and their adhesion to tissues to form clots
Scar-free	Dependent on its cationic properties. CS inhibits the production of type I collagen in wounds, promotes the production of granulation and epithelial tissue, as well as reducing wound contraction, thereby reducing scarring.

**Table 5:** Sources and characteristics of representative marine EPS.

Sources of EPS	Habitat	Functions and Applications	Refs
*Sphingobium yanoikuyae* BBL01	Coast	Gelling agent Metal-complexion Antioxidant	[220]
*Vibrio alginolyticus* 364	deep-sea	Anti-tumour	[223]
*Rhodothermus marinus* DSM 4252^T^	Shallow marine hot springs	Antioxidant Anti-haemolytic Anti-thrombotic	[224]
*Winogradsky* sp. CAL384 and *Shewanella* sp. CAL606	Antarctic Ocean	Emulsifier Chelates heavy metals	[225]
*Pseudomonas sp*. BGI-2	Glacier ice	Antioxidant Low temperature protection	[226]
*Paenibacillus* sp. TKU042	Marine chitinous materials	Antioxidant Anti-inflammatory Alpha-glucosidase inhibitor	[227]
*Bacillus subtilis* SH1	Marine surface sediment	Antiviral Antibacterial Antioxidant	[228]
*Bacillus vallismortis* WF4	Coast	Anti-fungal Anti-itch	[229]

**Table 6:** Summary of the raw materials and characteristics of MPs activities-enhanced wound dressings in recent years.

Bioactivities	Dressing Type	Structural Components	Active Agents	Other Features	Refs
Haemostatic Antibacterial	Hydrogels	Hydroxybutyl CS	Dopamine	Mussel-inspired technology High viscosity High mechanical strength Thermosensitive hydrogel	[238]
Haemostatic Antibacterial	Sponge	CS	Graphene-silver-polycationic peptide	--	[239]
Haemostatic	Hydrogels	Alginate Pept-1	Cross-linked zinc ions Tannic acid	High physical stability	[240]
Haemostatic	Hydrogels	Alginate GLE CMC	Cross-linked zinc ions Tannic acid	Effective drug delivery	[241]
Haemostatic	Sponge	CS	Tilapia peptides	--	[242]
Haemostatic Antibacterial Anti-inflammatory Promotes tissue regeneration	Sponge	Alginate CS Fucoidan	--	--	[144]
Haemostatic Promotes tissue regeneration	Sponge	CS PVA	--	For non-compression wounds	[243]
Antibacterial	Hydrogels	CS PVA	Ag NPs	--	[244]
Antibacterial Pro-regenerative Anti-inflammatory	Hydrogels	CS	AgNPs Nanocrystals	High physical stability Effective drug delivery	[245]
Antibacterial	Hydrogels	Alginate CaCO_3_ GDL	AgNPs	--	[246]
Antibacterial Anti-inflammatory	Hydrogels	Alginate Gum acacia	ZnNPs	--	[247]
Antibacterial	Hydrogels	CS Gelatin	Manuka honey	--	[248]
Antibacterial Anti-inflammatory	Hydrogels	Carboxylated Agarose	Zinc ions Tannic acid	pH-sensitive	[249]
Antibacterial Promotes tissue regeneration	Film	CS Modified bacterial cellulose	--	Self-healing High biocompatibility	[250]
Antibacterial	Film	CS Starch nanocrystals	Streptomycin	Sustained slow release	[251]
Antibacterial	Film	Alginate CaCO_3_	Oregano essential oil	High physical stability	[252]
Antibacterial	Membranes	CS Gelatin	Fe_3_O_4_ NPs	Extremely strong mechanical properties	[253]
Antibacterial	Nanofibres mats	Cellulose acetate	CS-Erythromycin NPs	High drug loading capacity High water holding capacity High porosity	[254]
Anti-inflammatory Promotes tissue regeneration	Hydrogels	QCS Matrigel Polyacrylamide	--	Good mechanical properties Good adhesion	[255]
Anti-inflammatory	Hydrogels	Alginate Polycaprolactone	Doxorubicin Ibuprofen	--	[256]
Anti-inflammatory	Films	CS	Cynara cardunculus leaves extracts	--	[257]
Anti-inflammatory	Membranes	CS PVA	Ibuprofen	Prepared by supercritical CO2 technology Highly biocompatible	[258]
Antioxidant Antibacterial Promotes tissue regeneration	Hydrogels	QCS-polyaniline Glycerol polyethylene glycol copolymer sebacate	--	Injectable Self-healing Adhesive conductive	[259]
Antioxidant Promotes tissue regeneration Anti-inflammatory	Hydrogels	Alginate PVA	Ag NPs hydroxymethylfurfural	--	[260]
Antioxidant Promotes tissue regeneration	Hydrogels	CS Heparin Poly(gamma-glutamic acid)	Superoxide dismutase	Good mechanical properties Adhesion	[261]
Antioxidant Antibacterial	Membranes	CS PVA	ZnO	Electrospun membrane	[262]
Antioxidant Promotes tissue regeneration	Nanofibres mats	Grafted CS Polypropylene carbonate	Curcumin	Sustained release	[263]
Haemostatic Anti-inflammatory Promotes tissue regeneration	Hydrogels	CMC PVA	--	Physically cross-linked Non-adhesive	[264]
Promotes tissue regeneration	Hydrogels	Ethylene glycol CS	GF VEGF PDGF-BB	Effective drug delivery Sustained release	[265]
Promotes tissue regeneration Antioxidant Antibacterial	Hydrogels	QCS Poly(N-isopropylacrylamide)	Reduced graphene oxide	Injectable Self-healing Self-contracting for wound healing Conductivity	[266]
Promotes tissue regeneration Haemostasis	Hydrogels	Alginate Adipic acid dihydrazide Polyglutamic acid	Bioglass	High physical stability	[267]
Promotes tissue regeneration	Hydrogels	CS PVA PCL	Heparin	Promotes angiogenesis	[268]
Promotes tissue regeneration	Hydrogels	Alginate	Borax	--	[269]
Promotes tissue regeneration Antibacterial	Membranes	CS Arginine CS	Arginine CS	Similar in structure to ECM Promotes cell adhesion Electrospun membrane	[270]
Promotes tissue regeneration	Hydrogels	Alginate Biological ceramics	Biological ceramics	Promotes angiogenesis High physical stability	[271]
Promotes tissue regeneration	Hydrogels	Alginate	Exosome	High physical stability High porosity	[272]
Scar-free ntibacterial	Hydrogels	CS PVP PEG	Tetracycline hydrochloride	Efficient drug delivery	[273]
Scar-free	Hydrogels	CMC	*Aloe vera*	*Aloe vera* synergistically enhances the scar-inhibiting activity of CMC	[58]
Scar-free Promotes tissue regeneration	Sponge/hydrogels	Rhizo CS	Platelet concentrates	Dressings healed wounds as functional tissue instead of scars	[274]
Scar-free Antibacterial	Membranes	CS Dextran Nanosoy Glycerol	*Aloe vera* Manuka Honey	--	[275]
Scar-free	Hydrogels	Alginate CS	AgNPs	High physical stability	[276]

**Table 7:** Summary of raw materials and characteristics of MPs hydrogels dressings in recent years.

Categories	Structural Components	Functional Components	Bioactivities	Other Features & Responsiveness	Refs
High mechanical properties	CMC Waterborne polyurethane—gelatine hydrolysate	--	Antibacterial	High mechanical strength Thermal stability	[348]
High mechanical properties	CS Poly (acrylamide)	Carbon nanotubes VEGF	Anti-inflammatory Promotes tissue regeneration	Double-network hydrogels High mechanical strength	[349]
Self-healing	Alginate Guar Gum	GA	Promotes tissue regeneration	Thermal stability High mechanical strength	[350]
Smart hydrogels	CS	Naproxen	In vivo anti-adhesion Analgesic	Thermosensitive Low side effects	[351]
Smart hydrogels	CS Methylenebisacrylamide	Red cabbage extract Curcumin	Not tested	pH-sensitive Dynamic monitoring of wound pH to assess wound recovery status by colourimetry Efficient drug delivery	[352]
Smart hydrogels	Dodecyl modified CS	Photothermolysis Ciprofloxacin	Strong, artificially controlled sterilisation Anti-inflammatory Antioxidants	Photosensitive Adherence Injectable	[353]
Injectable hydrogels	CMC Chondroitin oxide sulphate	Chondroitin oxide sulphate	Antibacterial Haemostatic	Longer gelation time Low cytotoxicity Self-healing	[291]
Injectable hydrogels	CS Oxidized konjac glucomannan	Ag NPs	Antibacterial	Self-adaptive Self-healing Adhesive	[354]
Injectable hydrogels	CS	bFGF Ag(crosslinked)	Antibacterial Anti-inflammatory Promotes tissue regeneration	Low cytotoxicity Promotes polarization of M2 macrophages	[355]
Injectable hydrogels	CS Bacterial cellulose	--	Antibacterial	Self-healing Enhanced mechanical properties	[356]
Injectable hydrogels	Alginate PVA	CaSO4	Promotes tissue regeneration	Effective drug delivery High mechanical strength	[357]
Mussel-inspired	CS Silk cellulose	Tannic acid (crosslinked)	Haemostasis	Strong wet tissue adhesion High mechanical strength	[241]
Mussel-inspired	CS Silk cellulose Dopamine reduced graphene oxide	Dopamine reduced graphene oxide	Antioxidant Promotes tissue regeneration	Strong wet tissue adhesion High mechanical strength Conductivity	[358]
Mussel-inspired	CS Gelatin graft-dopamine	Polydopamine-coated carbon nanotubes	Antibacterial Antioxidant Haemostasis Promotes tissue regeneration	Strong wet tissue adhesion High mechanical strength Conductivity Self-healing	[359]
Mussel-inspired	Alginate	Dopamine	Antibacterial	Strong wet tissue adhesion High mechanical strength	[360]
Mussel-inspired	Alginate nHA/PLGA-Dex	Schiff base	Promotes tissue regeneration Haemostatic	Strong wet tissue adhesion High mechanical strength	[361]

**Table 8:** Summary of raw materials and characteristics of MPs nanofibre dressings in recent years.

MPs Component	Other Main Components	Active Agents	Biological Activities	Other Features	Refs
CS	Polyvinylidene fluoride Polyhydroxybutyric acid	Gentamicin	Not tested	Double layer drug delivery Efficient drug delivery Strong mechanical properties	[390]
CS	PVA Starch	--	Antibacterial Promotes tissue regeneration	High water vapour transmission rate to provide a moist Well-oxygenated wound healing environment Low cytotoxicity	[391]
QCS	Collagen PCL PVA	--	Haemostatic, antibacterial Anti-inflammatory Promotes tissue regeneration	--	[392]
CS	PCL	Human granulocyte colony-stimulating factor-loaded CS NPs	Anti-inflammatory Promotes tissue regeneration	The stent promotes stem cell adhesion and proliferation, sustained slow release	[393]
CS	PCL PVA Polycaprolactone	Melatonin	Anti-inflammatory Promotes tissue regeneration	Three layers of nanofibres Hydrophilic effect	[394]
CS	PVA Carbopol Polycaprolactone	Curcumin Mesenchymal stem cells	--	Promotes tissue regeneration	[395]
Alginate	WPU CaCl	--	Not test	Effective drug delivery High mechanical strength	[396]
Alginate CS	Gentamicin	--	Antibacterial	Effective drug delivery Promotes tissue regeneration	[397]
Alginate	PUL	PL	Anti-inflammatory	High mechanical strength	[398]
Alginate	TOBC	Zn2+	Antibacterial	High mechanical strength	[399]
Alginate	PVA	Spider silks	Anti-inflammatory	Effective drug delivery Promotes tissue regeneration	[400]
Alginate CS	PCL Lumi	Doxycycline, PEO	Not test	Strong wet tissue adhesion High mechanical strength Effective drug delivery	[401]
Alginate CS	Glutaraldehyde polylysine	--	Promotes tissue regeneration	High water vapour transmission rate to provide a moist environment Effective drug delivery	[388]

**Table 9:** Summary of raw materials and characteristics of MPs membranes dressings in recent years.

Categories	Structural Components	Functional Components	Bioactivities	Other Features	Refs
Electrospun membranes	CS PCL	--	Promotes tissue regeneration	The ECM-like structure facilitates cell adhesion and penetration Promotes compartmentalization and prevents initial cell migration	[404]
Electrospun membranes	CS Cellulose Polyethylene oxide	Graphene	Antibacterial	Good water vapour transmission and breathability	[405]
Asymmetric membranes	CS PVP Nanocellulose	Stearic acid (coating)	Antibacterial	Unilateral hydrophobic Low cytotoxicity High biocompatibility	[406]
Asymmetric membranes	CS Gelatin methacrylate	Polycaprolactone Polylactic acid (dense layer)	Promotes tissue regeneration	Good mechanical properties Provide a moist environment for the wound healing Promotes cell adhesion Electrospun membranes	[407]
Asymmetric membranes	CS *Aloe vera*	Polycaprolactone(dense layer)	Promotes tissue regeneration	Good mechanical properties Promotes cell adhesion Electrospun membranes	[408]
Multi-layer membranes	CS Gelatine Poly(N-isopropylacrylamide)-grafted polyurethane	--	Promotes tissue regeneration	Provide a moist healing environment for the wound healing	[409]
Multi-layer membranes	Alginate CS	PMMA	Antibacterial Promotes tissue regeneration	Efficient drug delivery	[410]
Multi-layer membranes	Alginate CS	Genipin	Antioxidant	Good mechanical properties High water vapour transmission rate to provide a moist	[411]
Multi-layer membranes	Alginate	OBC	Antibacterial	Efficient drug delivery	[412]

**Table 10:** Summary of raw materials and characteristics of MPs-based sponge dressings in recent years.

MPs Composition	Other Main Components	Bioactivities	Other Features	Refs
CS Hydroxybutyl CS	--	Promotes tissue regeneration Antibacterial	Non-cytotoxic Highly absorbent	[422]
CS	HA, andrographolide lipid nanocarriers	Promotes tissue regeneration Scar-free	High encapsulation rate Slow release	[423]
CS	AgSD NPs	Antibacterial	Low cytotoxicity	[424]
CS	HA VEGF-loaded fibrin nanoparticles	Haemostasis Promote tissue regeneration	Proper mechanical properties	[425]
CS	GAGs Tranexamic acid	Haemostasis Promote tissue regeneration	Highly synergistic haemostatic	[426]
CS	Ag NPs Stearic acid (coating)	Antibacterial Promotes tissue regeneration	The presence of a hydrophobic An anti-adhesive surface allows the inside of the sponge to retain its water-absorbing capacity for a long time	[427]
Alginate	AV	Antibacterial	High degree of swelling	[428]
Alginate	1-ethyl-3-dimethyl aminopropyl carbon diimine hydrochloride	Promotes tissue regeneration	Good mechanical properties Considerable water vapour transmittance	[429]
Alginate	Graphene oxide	Promotes tissue regeneration	High flexibility and mechanical strength High water absorption	[430]
Alginate Fucoidan CS	--	Haemostasis Antibacterial Anti-inflammatory	Excellent elasticity Good mechanical properties	[144]
